# Genetic variability and natural selection at the ligand domain of the Duffy binding protein in brazilian *Plasmodium vivax *populations

**DOI:** 10.1186/1475-2875-9-334

**Published:** 2010-11-22

**Authors:** Taís N Sousa, Eduardo M Tarazona-Santos, Daniel J Wilson, Ana P Madureira, Paula RK Falcão, Cor JF Fontes, Luiz HS Gil, Marcelo U Ferreira, Luzia H Carvalho, Cristiana FA Brito

**Affiliations:** 1 Laboratory of Malaria, Centro de Pesquisa Rene Rachou/FIOCRUZ, Belo Horizonte, Brazil; 2 Department of General Biology, Universidade Federal de Minas Gerais, Belo Horizonte, Brazil; 3 Department of Human Genetics, University of Chicago, Chicago, USA; 4 Current Address: Nuffield Department of Medicine, Wellcome Trust Centre for Human Genetics, Headington, Oxford OX3 7BN, UK; 5 Universidade Federal de São João Del Rey, São João Del Rey, Minas Gerais, Brazil; 6 Laboratory of Bioinformatics, Empresa Brasileira de Pesquisa Agropecuária, Campinas, Brazil; 7 Department of Clinical Medicine, Universidade Federal do Mato Grosso, Cuiabá, Brazil; 8 Laboratory of Entomology, Instituto de Pesquisa em Patologias Tropicais - IPEPATRO, Porto Velho, Brazil; 9 Department of Parasitology, Institute of Biomedical Sciences, Universidade de São Paulo, São Paulo, Brazil

## Abstract

**Background:**

*Plasmodium vivax *malaria is a major public health challenge in Latin America, Asia and Oceania, with 130-435 million clinical cases per year worldwide. Invasion of host blood cells by *P. vivax *mainly depends on a type I membrane protein called Duffy binding protein (PvDBP). The erythrocyte-binding motif of PvDBP is a 170 amino-acid stretch located in its cysteine-rich region II (PvDBP_II_), which is the most variable segment of the protein.

**Methods:**

To test whether diversifying natural selection has shaped the nucleotide diversity of PvDBP_II _in Brazilian populations, this region was sequenced in 122 isolates from six different geographic areas. A Bayesian method was applied to test for the action of natural selection under a population genetic model that incorporates recombination. The analysis was integrated with a structural model of PvDBP_II_, and T- and B-cell epitopes were localized on the 3-D structure.

**Results:**

The results suggest that: (i) recombination plays an important role in determining the haplotype structure of PvDBP_II_, and (ii) PvDBP_II _appears to contain neutrally evolving codons as well as codons evolving under natural selection. Diversifying selection preferentially acts on sites identified as epitopes, particularly on amino acid residues 417, 419, and 424, which show strong linkage disequilibrium.

**Conclusions:**

This study shows that some polymorphisms of PvDBP_II _are present near the erythrocyte-binding domain and might serve to elude antibodies that inhibit cell invasion. Therefore, these polymorphisms should be taken into account when designing vaccines aimed at eliciting antibodies to inhibit erythrocyte invasion.

## Background

*Plasmodium vivax *malaria is a major public health challenge in Latin America, Asia and Oceania. Globally, 2.85 billion people are currently at risk of infection [1,2] and 130-435 million clinical cases are estimated annually worldwide [3]. In the Amazon Basin of Brazil, during the mid-1980s, *P. vivax *surpassed *Plasmodium falciparum *as the most frequent cause of clinical malaria, and currently *P. vivax *causes more than 400,000 malaria cases per year in Brazil [4].

Invasion of host blood cells by different species of malaria parasites depends on numerous receptor-ligand interactions. Most parasite proteins known to be involved in such interactions are highly polymorphic and are potential targets of naturally acquired immunity. However, relatively little is known about the patterns of polymorphisms in surface proteins of *P. vivax*. Merozoite invasion of erythrocytes by *P. vivax *relies on an interaction between a ligand on the parasite and the Duffy antigen/receptor for chemokines (DARC) on the surface of erythrocytes [5]. The parasitic ligand is a micronemal type I membrane protein called Duffy binding protein (DBP), or PvDBP in *P. vivax*. As demonstrated by a DBP gene-deletion experiment in *P. knowlesi *- a malaria parasite closely related to *P. vivax *that infects humans and other primates - DBP plays an important role in formation of the irreversible junction with erythrocytes, a key step of host cell invasion [6,7]. A series of competitive binding and directed-mutagenesis strategies have demonstrated the importance of the DBP/DARC interaction [5,7-9]. Moreover, individuals lacking Duffy receptors on their erythrocytes are highly resistant to *P. vivax *invasion [10,11]. Because antibodies against PvDBP inhibit the DBP/DARC interaction *in vitro *and also block the invasion of human erythrocytes [12-14], this protein is a major candidate for vaccines against *P. vivax*. Nevertheless, recent reports that *P. vivax *is able to infect and cause disease in Duffy-negative individuals suggest the existence of alternative invasion pathways yet to be elucidated [15-17].

The erythrocyte-binding motif of PvDBP is a 170 amino-acid stretch located in the cysteine-rich region II of the protein (PvDBP_II_). With 93% of PvDBP's polymorphic residues, PvDBP_II _is the most variable segment of the protein [18-20]. Although most of the cysteines and some of the aromatic residues in PvDBP_II _are involved in erythrocyte binding and are evolutionarily conserved [18,19,21,22], several non-synonymous polymorphic residues within PvDBP_II _have been identified in field isolates from Papua New Guinea [23,24], Colombia [25], South Korea [26], Brazil [27] and Thailand [28]. This observation is consistent with positive natural selection acting on PvDBP_II _and with allelic variation serving as a mechanism of immune evasion.

To formally test this hypothesis in a population genetics framework, several tests of neutrality have been applied to PvDBP_II _data (Tajima's D; Fu and Li's D and F; McDonald-Kreitman), and the rates of synonymous (*d*_*S*_) and non-synonymous (*d*_*N*_) substitutions have been compared based on the method of Nei and Gojobori (1986). However, these analyses have produced inconsistent results [29,30], and their assumptions are frequently inappropriate for the data analyzed. For instance, the Nei and Gojobori (1986) method of estimating *d*_*N *_and *d*_*S *_ignores either transition/transversion bias or unequal base/codon frequencies, which may lead to biased results [31]. On the other hand, phylogeny-dependent methods based on maximum likelihood estimates of *d*_*N *_and *d*_*S *_[31] assume no recombination and have been developed to be applied to sequences from independent, divergent species, rather than for intraspecific analysis [32]. In this study, these problems are overcome by a recently described probabilistic model called omegaMap [33] to infer whether diversifying natural selection has shaped the nucleotide diversity of PvDBP_II_. It uses a population genetics approximation to the coalescent with recombination (a natural model choice for population data), and it uses reversible-jump Markov Chain Monte Carlo (MCMC) to perform Bayesian inference on both the *d*_N_/*d*_S _ratio and the recombination rate, allowing each to vary along a DNA sequence. Being based on the Nielsen and Yang [34] model of codon evolution, omegaMap incorporates information about the transition/transversion ratio and codon frequencies from the data in a probabilistic framework. Specifically, the omegaMap approach was applied to PvDBP_II _to detect the action of diversifying natural selection on regions of PvDBP_II _containing B- and T-cell epitopes. This population genetics analysis was integrated with computational modelling of the 3-D structure of PvDBP_II_, which showed that several polymorphic residues (three of which are clearly under positive selection) map close to the erythrocyte-binding cluster of PvDBP_II_.

## Methods

### Parasite isolates and study sites

One hundred twenty-two field isolates of *Plasmodium vivax *were obtained from six sites across the Brazilian Amazon: Cuiabá (Mato Grosso State [MT], n = 25), Acrelândia (Acre State [AC], n = 25), Manaus (Amazonas State [AM], n = 20), Augusto Correa (Pará State [PA], n = 18), Porto Velho (Rondônia State [RO], n = 20), and Macapá (Amapá State [AP], n = 14) (see additional file 1: Locations of blood collection in Brazil). Epidemiological data indicate that malaria transmission in these areas is generally hypo- to meso-endemic [35-37]. The samples were collected from the different regions as follows: AC, between March and April 2004; AM, May 2003; AP, November 2004; PA, October 2005; RO, from October to December 2004; and MT, from July 2003 to July 2004. At the time of blood sample collection, the annual parasite index (API: the number of positive blood smears per thousand inhabitants) of the contagion localities allowed these areas to be classified as high risk (API >50: Augusto Correa/PA and Acrelândia/AC), medium risk (API 10 to 50: Manaus/AM) or low risk for malaria transmission (API <10: Macapá/AP) [38]. In these regions, the individuals were locally infected. However, in Cuiabá/MT and Porto Velho/RO, individuals were infected elsewhere, and therefore the risk of malaria transmission reported there was calculated based on the average API from contagion localities. Thus, both localities were classified as high-risk areas for malaria transmission.

### PvDBP_II _amplification and sequencing

Genomic DNA was immediately extracted from whole blood samples using the PUREGENE DNA isolation kit (Gentra Systems, Minneapolis, MN). The sequence encoding PvDBP_II_, corresponding to nucleotides 870 to 1545 (aa 290-515, reference sequence: Sal-I) [39], was amplified by PCR using high fidelity platinum Taq DNA polymerase (Invitrogen Corporation, Carlsbad, CA) and the primers forward: 5' ATGTATGAAGGAACTTACGAAT 3' and reverse: 5' ACCTGCCGTCTGAACCTTTT 3'. The PCR products were purified using a GFX-96 PCR kit (GE Healthcare, Little Chalfont, UK), and their sense and anti-sense strands were both sequenced using PCR primers and the DYEnamic ™ ET dye terminator kit (GE Healthcare). The products were analyzed on a MegaBace automated DNA sequencer (GE Healthcare). Since no heterozygous sequence variations were observed, it was inferred that these came from patients with a single infection or from the predominant variant of patients with multiple infections. All singletons were confirmed by sequencing from independent PCR amplifications. Six-fold sequence coverage was achieved for each of the analyzed isolates. The PvDBP_II _gene polymorphisms identified among the Brazilian Amazon isolates were compared to all DBP gene sequences of *P. vivax *in GenBank (last update on 25th February 2010). The PvDBP_II _sequences identified here were deposited in GenBank with accession numbers EU812839 to EU812960. Sequence alignments and comparisons were performed using the Clustal W multiple alignment algorithm in BioEdit Sequence Alignment editor using manual editing [40].

### Diversity analysis of PvDBP_II_

To estimate within-population diversity, the numbers of segregating sites and observed haplotypes were counted. Estimates of the nucleotide diversity (π), haplotype diversity (Hd), and their corresponding standard deviations (SDs) were obtained using DnaSP 4.10 software [41]. Between-population differentiation was measured using the pairwise fixation index *F*_ST _with Arlequin 3.01 software [42]. The *F*_ST _index (between-population component of the genetic variance, Wright 1951) was calculated assuming the Kimura 2-parameter mutation model [43,44]. The correlation between pairwise population genetic distances (*F*_ST_) and geographical distances was estimated by nonparametric Spearman's rank correlation.

### Analysis of recombination and linkage disequilibrium (LD)

The recombination parameter ρ among contiguous sites across PvDBP_II _was estimated by the method described by Li and Stephens [45] using PHASE 2.1 software. The parameter ρ, which can be estimated by different statistical methods, depends both on the recombination rate and on the effective population size, and therefore, it is proportional to the number of recombination events per generation that occurred in a population. The algorithm was run twice with 500,000 iterations, with a 20,000 iteration burn-in and a thinning interval of 1,000 iterations. The pairwise linkage disequilibrium (LD) across PvDBPII was analyzed using the r^2 ^statistic [46], and its significance was assessed by calculating LOD (base-10 logarithm of odds) scores. LD was calculated only for SNPs with a minor allele frequency (MAF) higher than 0.1 in the total Brazilian sample. These calculations were performed and visualized using the program Haploview [47].

### Tests of neutrality

The method developed by Wilson and McVean [33], implemented in the omegaMap software, was used to estimate the parameter ω = *d*_*N*_/*d*_*S*_, where *d*_*N *_is the rate of non-synonymous substitutions and *d*_S _is the rate of synonymous substitutions. Estimates of the parameter ω are indicators of the action of natural selection on coding sequences. Under no selection ω is expected to be equal to 1, under negative selection the value is expected to be significantly less than 1, and under positive selection the value is expected to be significantly greater than 1. This method employs a Bayesian approach to parameter estimation that is independent of phylogeny, and it is therefore less prone to falsely detecting diversifying selection. The model uses a population genetic approximation to the coalescent with recombination, and it uses reversible-jump Markov Chain Monte Carlo (MCMC) to perform Bayesian inferences of both ω and the recombination parameter ρ, allowing both parameters to vary along the sequence. In this study, analyses were conducted using 10 randomly chosen orderings of the haplotypes and the following priors: μ (synonymous transversion) = Improper inverse, κ (transition/transversion rate) = Improper inverse, ϕ (insertion/deletion rate) = Improper inverse. Since ω requires a proper prior, a log-normal distribution centered on log (ω) = 0 was used with a wide standard deviation of 2. An average block length of 20 codons was chosen to be around 10% of the sequence length. Estimates of the recombination parameter ρ were obtained by omegaMap among contiguous sites across PvDBP_II_. For ρ, there is no natural "reference" prior, so it was used an informative prior based on Gunasekera *et al*. [48]: a log-normal distribution with parameters log(0.1) and 1. As it was expected that there would be less information regarding ρ than ω, an average block length of 40 codons was assumed for ρ to impose greater smoothing. Sixteen independent MCMC chains were run for 90,000 iterations with a 10,000 iteration burn-in. The chains were compared for convergence and merged to obtain the posterior distributions. The results showed the geometric mean for the posterior distribution of ω and ρ and the 95% HPD (highest posterior density) interval. To determine the influence of the choice of priors on the posteriors, the analyses were repeated with an alternative set of priors (μ = Exponential mean 0.02; κ = Exponential mean 2.0; ϕ = Exponential mean 0.1; ω = Exponential mean 1.0; ρ = Exponential Mean 0.14). The means for priors for μ, κ and ρ were chosen using the posterior distributions generated with the first prior set. A mean value of 1 was used for the ω prior based on the null hypothesis of selective neutrality.

To integrate the evolutionary analysis with structural information, the posterior probability of selection at each codon was mapped onto the 3-dimensional structure of PvDBP_II _(see below) to visualize the spatial distribution of selected sites. The correlation between *F*_ST _for each polymorphism and its ω estimators was calculated using Pearson's correlation (Minitab statistical software, release 13.20).

### Prediction of promiscuous T-cell epitopes

Because only a few epitopes have been described for PvDBP [30,49], an *in silico *analysis was used to infer new and promiscuous PvDBP_II _T-cell epitopes able to bind to several alleles of host MHC molecules. To identify the MHC Class-I binding regions in PvDBP_II_, the ProPred-I program was used [50]. This program uses matrices for each of the 47 MHC Class-I alleles the elements of which denote the affinity of antigen amino acids for specific MHC alleles, thereby allowing peptides in PvDBP_II _with antigenic properties to be identified. Quantitative matrices derived from the published literature [51] and implemented in ProPred were also used to predict MHC Class-II binding regions in PvDBP_II _[52].

### 3-D structural model of PvDBP_II_

Homology modelling was performed with Modeller 8v2 software using the X-ray structure of the *Plasmodium knowlesi *DBL domain as a template (Pkα-DBL) (PDB file: 2C6J, chain A) [53]. This template shares 71% sequence identity with PvDBP_II_. The 3-D structure of PvDBP_II _was modelled between amino acids 261 and 553. The quality of these models was evaluated with PROCHECK v.3.5.4 software [54]. Superpositioning and renderings were carried out with PyMol v1.0 software [55].

## Results

### Polymorphisms in PvDBP_II _among Brazilian Amazon isolates

**Figure 1:**
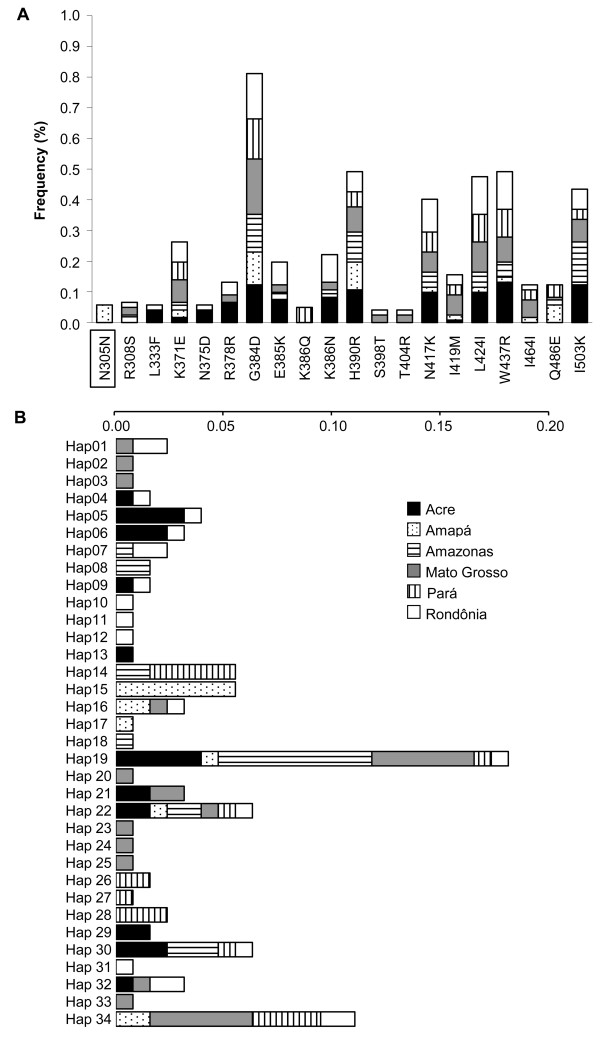
**Variability in PvDBP**_**II**_. (A) Frequencies of polymorphic amino acid residues for each Brazilian geographic region. The first letter indicates the more prevalent amino acid and the last letter the polymorphic amino acid at that position (position number in the middle). The amino acid positions are consistent with Fang *et al*. [39]. The box indicates a substitution exclusive to Brazilian isolates. (B) Haplotype frequencies of PvDBP_II _between Brazilian regions. The scale bar shows the frequency of each haplotype per region indicated in the horizontal boxes.

By sequencing 675 bp of the PvDBP region II of 122 isolates from six Brazilian Amazon regions, 19 polymorphic sites (three synonymous substitutions and 16 non-synonymous substitutions) were identified with an overall nucleotide diversity of 0.0081 (Table 1 and Figure 1A). Of 16 sites with non-synonymous polymorphisms, one site at position 386 was not dimorphic. At this site, lysine could be substituted by glutamine or asparagine (K386Q/N). A positive correlation was found between the risk of malaria transmission in each area (estimated based on the annual parasitic index [API]) and both the nucleotide diversity and the haplotype diversity of PvDBP_II _(Spearman correlation coefficients: *r*_*s *_= 0.811, *p *= 0.049; *r*_*s *_= 0.828, *p *= 0.041, respectively). Most of the non-synonymous polymorphisms were non-conservative (75%) with changes of the physico-chemical family of the respective amino acid. In a comparison of the polymorphisms identified in isolates studied here to the 401 PvDBP_II _sequences available in GenBank, only one was recognized as exclusive to Brazilian isolates: N305N (a synonymous substitution of Asparagine at position 305), and this was present in 10 isolates from Amapá (see additional file 2: Table with description of PvDBP_II _polymorphisms identified in Brazilian isolates).

**Table 1:** Nucleotide and haplotype diversity of PvDBPII gene and FST among Brazilian populations

					***F***_**ST **_**(×100)**^**f**^				
					
**Population**^**a**^	**S**^**b**^	**π**^**c **^**(SD)**	**H**^**d**^	**Hd**^**e **^**(SD)**	AP	AM	PA	RO	MT
AP (N = 14)	11	0.0050 (0.0013)	6	0.747 (0.111)	-				
AM (N = 20)	11	0.0057 (0.0009)	7	0.779 (0.082)	21.02*	-			
PA (N = 18)	11	0.0070 (0.0004)	8	0.869 (0.049)	18.68*	15.56*	-		
RO (N = 20)	17	0.0087 (0.0008)	16	0.979 (0.021)	30.29*	16.87*	11.02*	-	
MT (N = 25)	16	0.0080 (0.0009)	14	0.897 (0.043)	14.51*	6.97	2.33	7.18*	-
AC (N = 25)	13	0.0079 (0.0005)	11	0.917 (0.028)	26.59*	7.24*	14.68*	3.94	9.31*

Total/mean (N = 122)	19	0.0081 (0.0003)	34	0.934 (0.012)					

Populations from Amapa (**AP**), Amazonas (**AM**), Para (**PA**), Rondonia (**RO**), Mato Grosso (**MT**) and Acre (**AC**). **N**; number of isolates, **S; **Number of segregating (polymorphic/variable) sites; **K; **Average number of pairwise nucleotide differences, **H; **Number of haplotypes, **Hd; **Haplotype diversity; π; Observed average pairwise nucleotide diversity, ***F***_**ST**_; Fixation index, a measure of genetic differentiation between populations. * *p *< 0.05

The polymorphisms observed in Brazil were arranged in 34 haplotypes (Figure 1B), corresponding to a mean haplotype diversity of 0.934 (Table 1). The modal haplotype Hap19 was observed in all six sampled regions. The *F*_ST _values of the populations ranged from 2.33 to 30.29%, with the highest values corresponding to the Amapá population (Table 1). Moreover, the *F*_ST _values did not correlate with the geographic distance between populations (Spearman correlation coefficient *r*_*s *_= 0.0750, *p *= 0.7905).

### Intragenic recombination and linkage disequilibrium in PvDBP_II_

**Figure 2:**
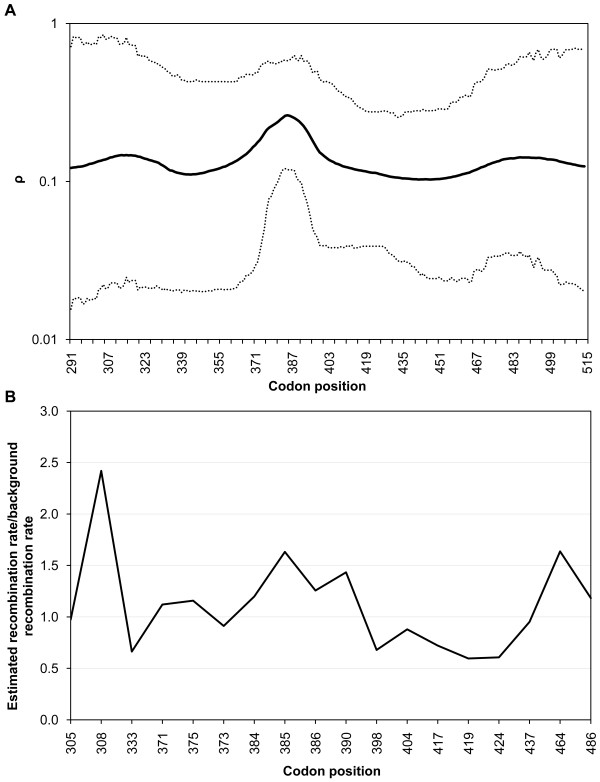
**Spatial variation in recombination across PvDBP**_**II**_. (A) Recombination parameter estimates (ρ) calculated using omegaMap. ρ > 0.1 is taken as evidence of recombination. The sitewise mean (solid line) and 95% HPD (highest posterior density) interval (dotted lines) are shown. (B) The factor by which the recombination rate between adjacent loci exceeds the background rate (ρ) as estimated using PHASE software.

**Figure 3:**
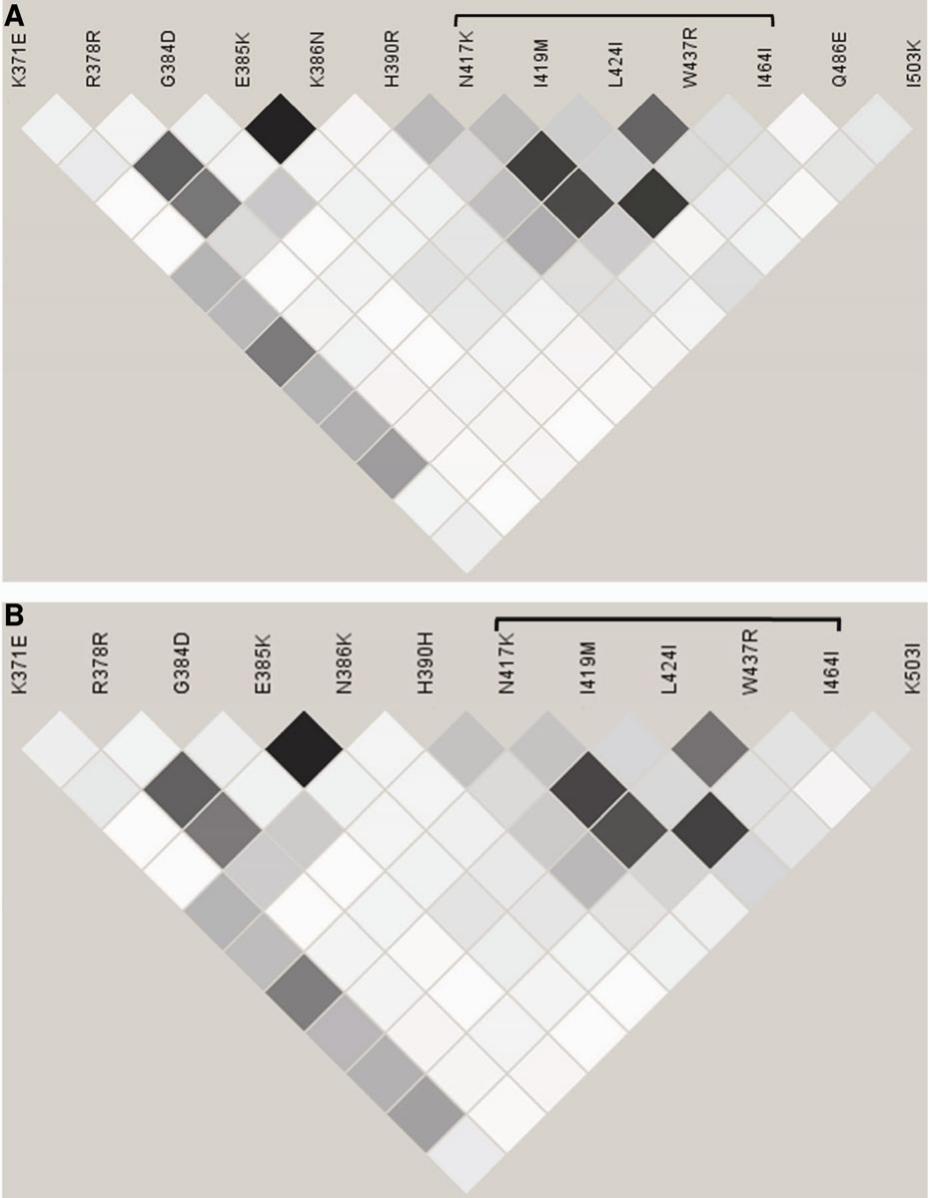
**Linkage disequilibrium in PvDBP**_**II.**_. Patterns of pairwise linkage disequilibrium (*r*^2^) in the PvDBP_II_-encoding gene from Brazilian isolates. (A) Linkage disequilibrium was calculated for SNPs for which the frequency of the minor allele was at least 0.1 in the entire sample. White: *r*^*2 *^= 0; black: *r*^*2 *^= 1; shades of gray: 0 <*r*^*2 *^< 1. The bracket indicates the cluster of amino acids with the highest *r*^*2*^. (B) LD was calculated as previously described but not including the Amapá population.

In the absence of recombination and recurrent mutation, it would be expected that the 19 polymorphic sites observed would be arranged into a maximum of 20 haplotypes. Because 34 haplotypes were detected, it was inferred that recombination (and possibly recurrent mutation) has shaped the pattern of PvDBP_II _variation in Brazilian populations. Figure 2A shows the variation in the recombination parameter (ρ) (calculated using the software omegaMap) along the PvDBP_II _sequence, indicating that at least one region was clearly affected by recombination (aa 381-390, lower 95% HPD > 0.1). These results were further confirmed by estimating the recombination parameter with PHASE 2.1 software (Figure 2B) and also by identifying regions with low LD across PvDBP_II _using the statistic r^2 ^measured with Haploview (Figure 3A).

Consistent with the observed haplotype structure and ρ estimates, linkage disequilibrium (LD) across the PvDBP_II _sequence is low in the Brazilian isolates (Figure 3A). In fact, an analysis of the 12 SNPs in the whole sample with a minor allele frequency higher than 0.1 showed that only 8 of 66 pairwise combinations showed r^2 ^> 0.50. The highest r^2 ^values were observed for several spatially close polymorphisms, encompassing the SNPs delimited by N417K and I464I (LOD scores always > 6.64). In particular, three residues within this set were spatially close in the 3-D model (417, 419 and 424). Because the LD analysis could be affected by population structure, the data analysis was repeated without the Amapá population (the most differentiated population according to *F*_ST _value), and an almost identical LD pattern was observed (Figure 3B).

### Evidence of natural selection on PvDBP_II _and its epitopes

**Figure 4:**
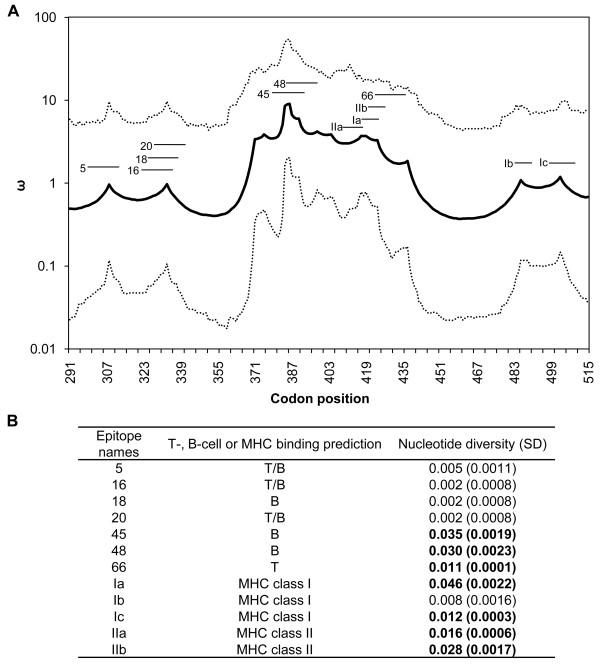
**Spatial variation in selection parameters across PvDBP**_**II**_. Omega parameter estimates (ω) calculated using omegaMap. ω > 1 is evidence for diversifying natural selection, whereas ω < 1 is evidence for purifying natural selection. The sitewise mean (solid line) and 95% HPD intervals (dotted lines) are shown (A). The short lines above the solid line represent previously identified epitopes: 5 [299-VNNTDTNFH(R/S)DITFR-313], 16 [321-LIYDAAVEGDLL(L/F)KL-335], 18 [325-AAVEGDLL(L/F)KLNNYR-339], 20 [329-GDLL(L/F)KLNNYRYNKD-343], 45 [379-SIFGT(D/G)(E/K)(K/N)AQQ(R/H)RKQ-393], 48 [385-(E/K)(K/N)AQQ(R/H)RKQWWNESK-399] and 66 [421-ICK(L/I)NV(A/P)VNIEPQIY-435] [30,49] and *in silico *predicted promiscuous epitopes: Ia [416-G(N/K)F(I/M)WICK(L/I)-424], Ib [482-KSYD(Q/E)WITR-490], Ic [497-VLSNKF(I/K)SVKNAEK-510], IIa [408-YSVKKRLKG(N/K)-417] and IIb [418-F(I/M)WICK(L/I)NV-426]. (B) Nucleotide diversity (π) and standard deviation (SD) were calculated for each epitope sequence. Epitope nucleotide diversity values significantly higher than the global nucleotide diversity of PvDBP_II _(π = 0.0081) are shown in bold.

To gain insight into the action of natural selection, the omegaMap approach was used [33], which is suitable for genomic regions (such as the PvDBP_II _gene) where recombination prevents the reliable reconstruction of a phylogeny. This analysis, which estimates ω and its associated HPD interval, also revealed that natural selection does not act uniformly within the gene (Figure 4A). The sequence contains regions where purifying selection seems to predominate, regions with ω values close to 1 that appear to be neutral or less affected by selection and regions under positive natural selection (ω >1), which are mainly in a central region of the protein (aa 368-439). To determine the influence of the choice of priors on the posteriors, the analyses were repeated with an alternative set of priors. Estimates for ρ and ω were qualitatively similar for the two sets of priors used (see additional file 3: Recombination and selection parameters in PvDBP_II _with an alternative set of priors).

Pathogen epitopes are responsible for host immune recognition. Thus, the association between positive natural selection and the presence of T- and B-cell epitopes in PvDBP_II _was tested (Figure 4A). The results indicated that ω was higher for sites belonging to B-cell and T-cell epitopes (experimentally identified and predicted) than for non-epitope sites (Mann Whitney U = 2587.00, *p *< 0.0001) [30,49]. The present study therefore showed that positive natural selection preferentially acts on epitopes in PvDBP_II_. In addition, the epitopes in the region showing the highest ω values also have greater nucleotide diversity (0.011 - 0.046) than the value for all of PvDBP_II _(0.0081) (Figure 4B). In particular, nucleotide diversity was the highest in *in silico *predicted epitope Ia. Ia contains three spatially proximate polymorphic residues at positions 417, 419 and 424 and shows a clear signature of positive natural selection, with ω values of 3.72, 3.72 and 3.31, respectively.

### Spatial distribution of polymorphisms across PvDBP_II_

**Figure 5:**
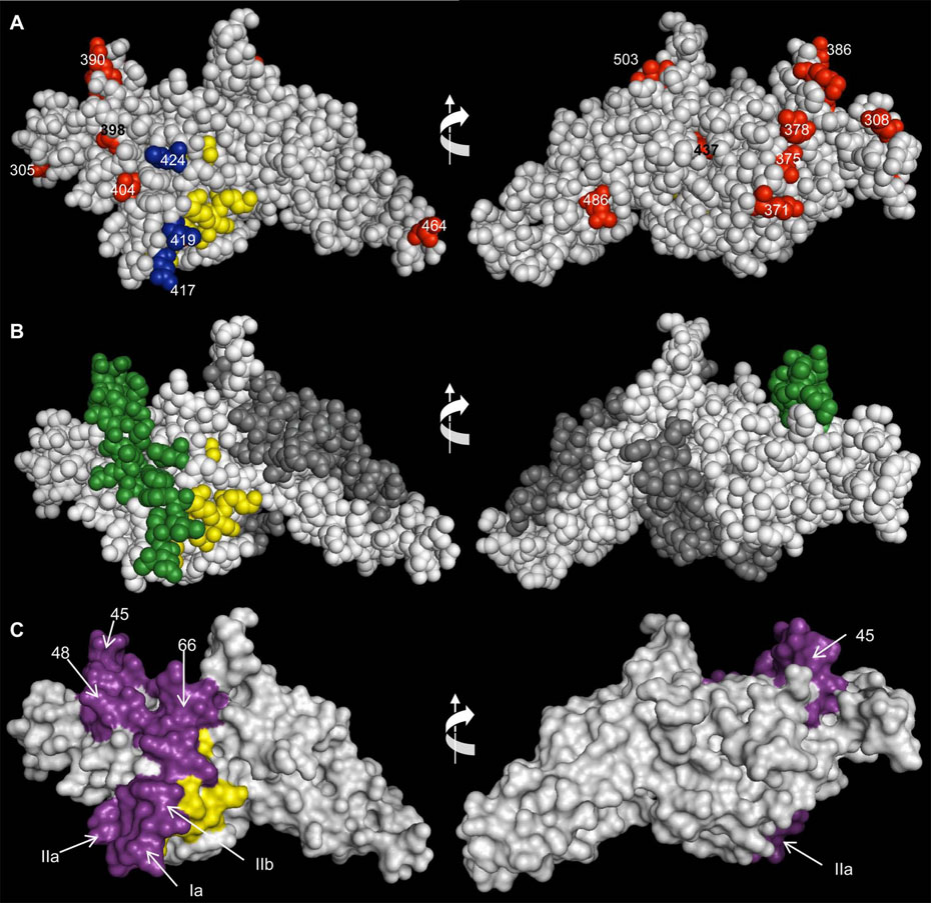
**3-D structure of PvDBP**_**II**_. Space-filling model of the 3-D structure PvDBP_II _showing the DARC-binding residues (Tyr 94, Asn 95, Lys 96, Arg 103, Leu 168 and Ile 175 - yellow) [53]. (A) Polymorphic residues identified in the Brazilian isolates are shown in blue or red for residues close to or not close to the DARC-binding domain, respectively. All positions of these residues on the sequence are indicated. (B) Residues on the 3-D structure are color coded according to the posterior probability of positive selection as calculated using omegaMap. Green residues indicate high posterior probability (HPD > 93%) of positive selection. Codons that seem to be less affected by natural positive selection (HPD < 93%) are colored in white. Residues in dark gray were not analyzed by omegaMap. (C) The previously identified epitopes 45, 48, and 66 [30,49] and the *in silico *predicted T-cell epitopes IIb, IIa and Ia are shown in violet. The model's rotation in the vertical plane is indicated by arrows.

The crystallographic structure of the Pkα-DBL binding-domain was used to produce a computational model of PvDBP_II_. In Figure 5A, this model of PvDBP_II _is presented in space-filling form, and it shows the previously identified essential, invariant and surface-exposed contact residues required for the recognition of DARC on human erythrocytes [53], as well as the residues that are polymorphic in the Brazilian Amazon samples. Although polymorphic residues were widely distributed throughout the sequence, the polymorphic residues at positions 417, 419, and 424, which are part of a block of high LD, are close to the erythrocyte-binding cluster of PvDBP_II _(Figure 5A). Both K417 and I424 are prevalent in Brazil as well as in other studied areas (Additional file 2).

The three-dimensional model of PvDBP_II _also shows the residues according to the posterior probability of their estimated ω (Figure 5B). Residues that seem to be less affected by positive selection (shown in white) were distributed throughout the protein, and interestingly, residues associated with high ω (likely to have evolved under positive selection, shown in green) were close to (and on the same face of) the proposed DARC-recognition site. Moreover, these residues tend to be in epitope regions, which were surface exposed (Figure 5C).

## Discussion

The data obtained in this study regarding the nucleotide diversity of PvDBP_II _among 122 *P. vivax *isolates from Brazil provide evidence that (i) recombination plays an important role in determining the haplotype structure of PvDBP_II_; and (ii) natural selection acts differentially across the PvDBP_II _sequence. Indeed, the gene appears to contain neutrally evolving codons as well as codons evolving under natural selection, and these latter codons are associated with high ω values (diversifying selection). The most striking conclusion of the current study is that diversifying natural selection acts on one PvDBP_II _region containing B- and T-cell epitopes previously identified and predicted *in silico *[30,49]. In particular, the amino acid residues at positions 417, 419, and 424 are associated with high ω values and map close to the erythrocyte-binding cluster of the molecule.

Diversifying selection has been observed in multiple proteins involved in parasite-host interactions, and it is generally attributed to the selective pressure of the host's immune response [56]. This study inferred that the evolution of the ligand domain of PvDBP, particularly of the codons for epitopes 45, 48, 66, Ia, IIa and IIb, is driven by diversifying selection (as indicated by high ω values). These epitopes are surface exposed and have significantly higher nucleotide diversity than the other residues in this region. However, inferential methods can lack the statistical power to reject neutrality and detect the action of diversifying selection, particularly when the analysis is restricted to a few codons such as epitope sequences. In the present study, this may be the case for epitopes 5, 16, 18, 20, Ib and Ic. In this context, the use of appropriate methods for inferring natural selection is important, and new methods based on more realistic assumptions about molecular evolution (such as that of Wilson-McVean) will help to assess the role of diversifying selection in parasite diversity.

### Comparisons with previous analyses

The low linkage disequilibrium (LD) and high recombination parameter across the PvDBP_II _gene suggest that meiotic recombination partially accounts for the observed haplotype diversity. This result corroborates the findings of Martinez *et al*. [29], who also found high recombination rates in PvDBP_II _among isolates from Papua New Guinea, Colombia and Korea. Using omegaMap with PNG sequences, similar results were obtained (see additional file 4: Spatial variation in recombination (ρ) and omega parameter (ω) calculated using omegaMap software among Papuan New Guinea PvDBP_II _sequences). However, Cole-Tobian and King [30] found a low recombination rate in PvDBP_II_, but the test used (Sawyer's permutation test) is not appropriate for estimating the recombination rate in relatively short regions like this [57]. The results presented here are supported by three different analyses: Haploview; PHASE and omegaMap. These results reinforce the importance of recombination as well as natural selection in the generation and maintenance of the genetic diversity of PvDBP_II_. Multiple recombination events have also been described in other *P. vivax *antigens, such as the Apical Membrane Antigen 1 (PvAMA1), Merozoite Surface Protein-3α (MSP) and MSP-1 [48,58,59].

Previous attempts to infer the action of natural selection in PvDBP_II _with different neutrality tests yielded inconclusive results. Martinez *et al*. [29] used Tajima's [60] and Fu-Li's [61] neutrality tests and found an excess of rare alleles for PvDBP_II_, which is consistent with either the action of a selective sweep or the purifying natural selection (as suggested by the authors). A limitation of these tests is that the allelic spectrum is influenced both by natural selection and by the demographic history of the population, and it is not trivial to discriminate between the influence of these two variables without significant knowledge of the demographic history of *P. vivax *populations, which is not the case. A second type of neutrality test applied to the PvDBP_II _data compares the pattern of synonymous (assumed to be neutral) and non-synonymous polymorphisms (for which natural selection is possible). Because the demographic history of a population affects synonymous and non-synonymous sites equally, these tests are less sensitive to the potential confounding effects of the demographic history of populations. Applications of these analyses to PvDBP_II _data include the McDonald-Kreitman test [62] and comparisons between *d*_*N *_and *d*_*S *_estimated using the Nei-Gojobori method [63], and these suggest the action of positive natural selection acting on PvDBP_II _[29,30]_. _However, the new method of Wilson-McVean [33] has enabled us to map how the action of natural selection varies across PvDBP_II _and to integrate this evolutionary information with an epitope analysis and structural modelling. Indeed, the Wilson-McVean method has four features that improve its performance in correctly identifying the effect of diversifying selection. First, it assumes a more realistic mutational model [34] that allows for different rates of transitions and transversions and for differences in codon frequencies. Second, its fully Bayesian approach allows ω parameters and their associated HPD intervals to be estimated, providing a measurement of uncertainty. Third (and more importantly), the Wilson-McVean method uses a reversible-jump MCMC to explore the posterior distribution of ω, allowing it to vary along the length of the sequence. Although incorporating these more realistic assumptions implies a computational cost, the analyses have demonstrated their value. The Wilson-McVean method allowed us to map how diversifying selection has acted across PvDBP_II _and to test the association between the presence of epitopes and the action of natural selection. Last (but not least), recombination, which has been important in shaping PvDBP_II _diversity, does not affect the performance of Wilson-McVean estimations. These features, the fact that a highly polymorphic and functionally important region of PvDBP was examined for which results of functional studies are available, and the complementary structural and epitopes analyses partially overcome one of the limitations of this study: the small size of the studied region. A thorough examination of the entire *PvDBP *sequence in different populations will provide a better understanding of how natural selection has shaped the different regions of the gene across different populations, including Brazil. However, on the basis of the results presented here, it is anticipated that, if the observed level of recombination affects the entire gene, signatures of natural selection might not propagate across large genomic regions. Therefore, population genetics methods that are able to examine signatures of selection at localized regions may be more appropriate for an evolutionary analysis of PvDBP.

### Effects of population structure

In the present study a high level of population structure was observed, as indicated by high *F*_ST _values (from 2.33 to 30.29%), and this could affect the estimates of both linkage disequilibrium and ω. However, when the most differentiated population (Amapá) was excluded from the analyses, the same LD pattern was still observed, which indicates that these results are not an artifact of population structure. In particular, the residues at positions 417, 419, and 424, which are associated with high ω values and map near the DARC binding domain in the structural model, are in LD even when the Amapá population is excluded from the analysis.

The possibility that the high ω values inferred could be an artifact of population structure was also investigated. Two features of the data suggest that this is not the case. First, if there is more differentiation (*F*_ST_) of non-synonymous polymorphisms than synonymous polymorphisms, high ω values may emerge when ω is estimated with sequences from different clustered populations, but this is not the case in the data presented here (non-synonymous *F*_ST: _14.7%, synonymous *F*_ST_: 24.0%). Second, if population structure creates high ω values, a correlation between the *F*_ST _for non-synonymous sites and the ω values of the codons containing these sites is expected, but this was not observed (Pearson correlation 0.2647, *p *= 0.303). Therefore, there is no evidence that the observed pattern of LD and inferences about positive natural selection are artifacts of the population structure of our Brazilian samples.

### Immunological implications

*Plasmodium vivax *immune evasion could be explained by a model based on a "just-in-time" release mechanism of PvDBP [53,64]. This model proposes that DBP is released from micronemes immediately before binding to DARCs, thereby avoiding immune inhibition by direct binding of antibodies to PvDBP. Therefore, residues on the surface of the protein that is opposite to that of the erythrocyte-binding domain remain exposed and are immune system targets. Supporting this model is a previous observation that polymorphic residues occur on the opposite side from the erythrocyte-binding domain [53]. Therefore, from the perspective of population genetics, it may be expected that positive natural selection imposed by the host's immune response would preferentially act on the protein surface opposite from the erythrocyte-binding domain, and values of ω > 1 would be concentrated on this side of the protein. Because the results presented here do not match this expectation (Figure 5), the analysis of the Brazilian isolates does not support the "just-in-time" release model. Notably, the results presented here show that stretches with high genetic diversity and residues under positive selection are present near the erythrocyte-binding domain. These results would be expected under a model of immune escape, in which polymorphic residues near the binding site elude recognition by inhibitory antibodies, implying that positive selection acts preferentially on residues near the erythrocyte binding site. This model is different from the "just-in-time" model but is not mutually exclusive.

In fact, the polymorphic residues at positions 417, 419 and 424, which form a unit of LD and are associated with high ω values, map to near the erythrocyte-binding domain. The residue at position 417 was also reported to be part of a linked haplotype in PNG (N417K, W437R, I503K) [9]. This model of immune evasion has been proposed for PvDBP [24,65-67] and also for PfAMA1 [68]. Recent functional results from Grimberg *et al*. [13] support this mechanism and show that antibodies involved in invasion-inhibitory activity also block PvDBP-DARC interaction. Recently, it was demonstrated that some epitopes described here (epitopes Ia and IIb) were also recognized by inhibitory antibodies in immune sera of individuals from Papua New Guinea as linear B-cell epitopes [69]. These epitopes include residues 417, 419 and 424, suggesting their involvement as targets of inhibitory antibodies.

Recent data from experimental and natural infections support our findings that PvDBP polymorphisms might have immunological relevance. Using rabbit immune sera, VanBuskirk *et al*. [9] demonstrated that polymorphisms change the antigenic character of DBP and can compromise immune inhibition. Moreover, evidence was found that in the Amazon area, long-term exposure to malaria is required to allow the production of anti-DBP antibodies able to inhibit the binding of different PvDBP_II _variants to DARC [12]. These results support the hypothesis that immune responses against PvDBP are haplotype-dependent, as has also been proposed for the apical membrane antigen 1 of *P. falciparum *(PfAMA1), another micronemal protein vaccine candidate [70]. However, neutrality tests simply test the hypothesis that the observed pattern of diversity cannot be explained merely by the neutral model of evolution, and may suggest which type of natural selection has acted. Inferences about natural selection need to be complemented with experimental tests of the effects of the observed variations on parasite invasion and immune escape.

## Conclusions

The low LD and the high recombination rates observed in PvDBP_II _haplotypes suggest that recombination and diversifying selection are major factors influencing PvDBP_II _haplotype diversity in the Brazilian Amazon region. The results presented here stress the importance of using appropriate tests (such as the omegaMap approach [33]) to infer the relative roles of natural selection and recombination when determining the pattern of diversity of parasite genes. These genes are often involved in interactions with host immune systems, and they are strong candidates for involvement in co-evolutionary processes. However, all methods of inferring the action of positive selection have limitations [31,33,71]. Therefore, as pointed out by Nozawa *et al*. [71], it is important to complement (as in this study) pure population genetics inferences with information from structural modelling, *in-silico *and experimental inferences about epitopes, and functional studies. Taken together, these results suggest that vaccine strategies based on inhibitory anti-DBP antibodies that take into account PvDBP_II _haplotype variability can overcome the problem of antigen variability and generate a strain-transcending and long-lived vaccine. Moreover, the recent finding of Duffy-negative individuals infected with *P. vivax *emphasizes the importance of a multivalent vaccine strategy that, in addition to eliciting inhibitory antibodies, may also reduce the ability of parasite strains to escape immunological control.

## Competing interests

The authors declare that they have no competing interests.

## Authors' contributions

TNS performed all the experiments and analyzed the data. APM supervised the statistical analysis. DJW managed the omegaMap analysis. PRKF supervised the modelling experiments. CJFF, JMS, AARD'AC, MUF and LHSG organized and supervised the field work. LHC and CFAB designed the study. CFAB and ETS supervised the data analysis. TNS, CFAB and ETS wrote the paper with critical suggestions from LHC and MUF. All authors read and approved the final manuscript.

## Supplementary Material

Additional file 1 **Locations of blood collection in Brazil**. The cities are indicated with their respective states: Amazonas (AM), Pará (PA), Amapá (AP), Acre (AC), Rondônia (RO) and Mato Grosso (MT). The areas where malaria is endemic are shown in gray. Click here for file

Additional file 2 **PvDBP_II _polymorphisms of Brazilian isolates**. This file provides descriptions of the PvDBP_II _polymorphisms identified in the Brazilian isolates by geographic region as well as polymorphisms that were previously described in other localities. Click here for file

Additional file 3 **Recombination and selection parameters in PvDBP_II _with an alternative set of priors**. The spatial variation in recombination and selection parameters across PvDBP_II _was obtained using omegaMap software. The analysis was performed using an alternative set of priors: μ = Exponential mean 0.02; κ = Exponential mean 2.0; ϕ = Exponential mean 0.1; ω = Exponential mean 1.0; ρ = Exponential Mean 0.14. The means for the priors for μ, κ and ρ were chosen using the posterior distributions generated with the first prior set. (A) Recombination parameter estimates (ρ). The sitewise mean (solid line) and 95% HPD (highest posterior density) interval (dotted lines) are shown. (B) Omega parameter estimates (ω). The sitewise mean (solid line) and 95% HPD intervals (dotted lines) are shown. Click here for file

Additional file 4 **Spatial variation in recombination (ρ) and omega (ω) among Papuan New Guinea PvDBP_II _sequences calculated using omegaMap**. (A) Evidence of recombination is ρ > 0.1. (B) Evidence of diversifying natural selection is ω > 1 and for purifying natural selection it is ω < 1. The sitewise mean (solid line) and 95% HPD intervals (dotted lines) are shown. Click here for file
