# Supplementary figures and images for: Genetic variability and natural selection at the ligand domain of the Duffy binding protein in brazilian Plasmodium vivax populations
Source: Malar J. 2010 Nov 22;9:334. doi: 10.1186/1475-2875-9-334 (PMC3003673; doi:10.1186/1475-2875-9-334)

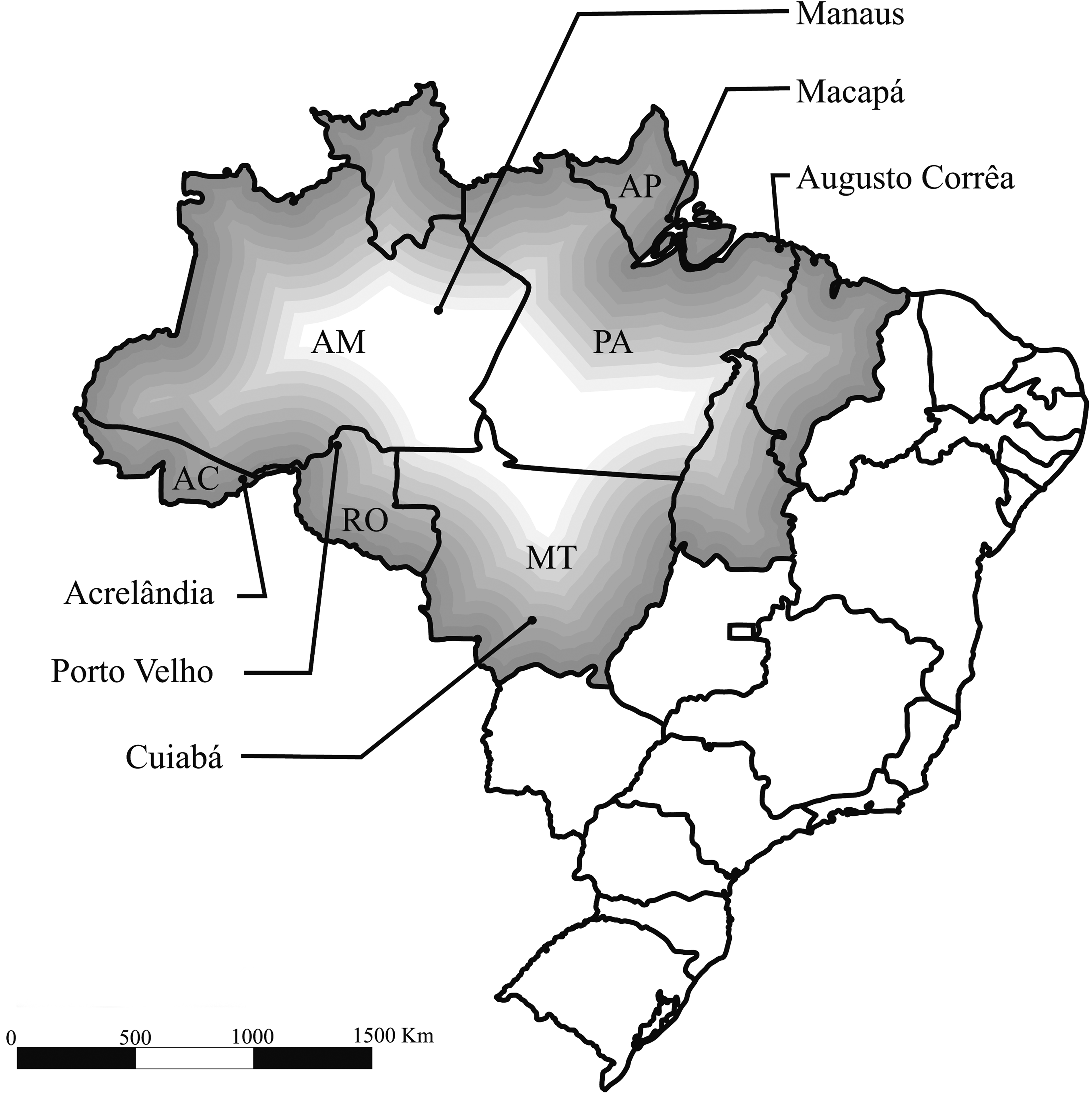

Supplement: Additional file 1 — Locations of blood collection in Brazil. The cities are indicated with their respective states: Amazonas (AM), Pará (PA), Amapá (AP), Acre (AC), Rondônia (RO) and Mato Grosso (MT). The areas where malaria is endemic are shown in gray. [file 1475-2875-9-334-S1.TIFF]

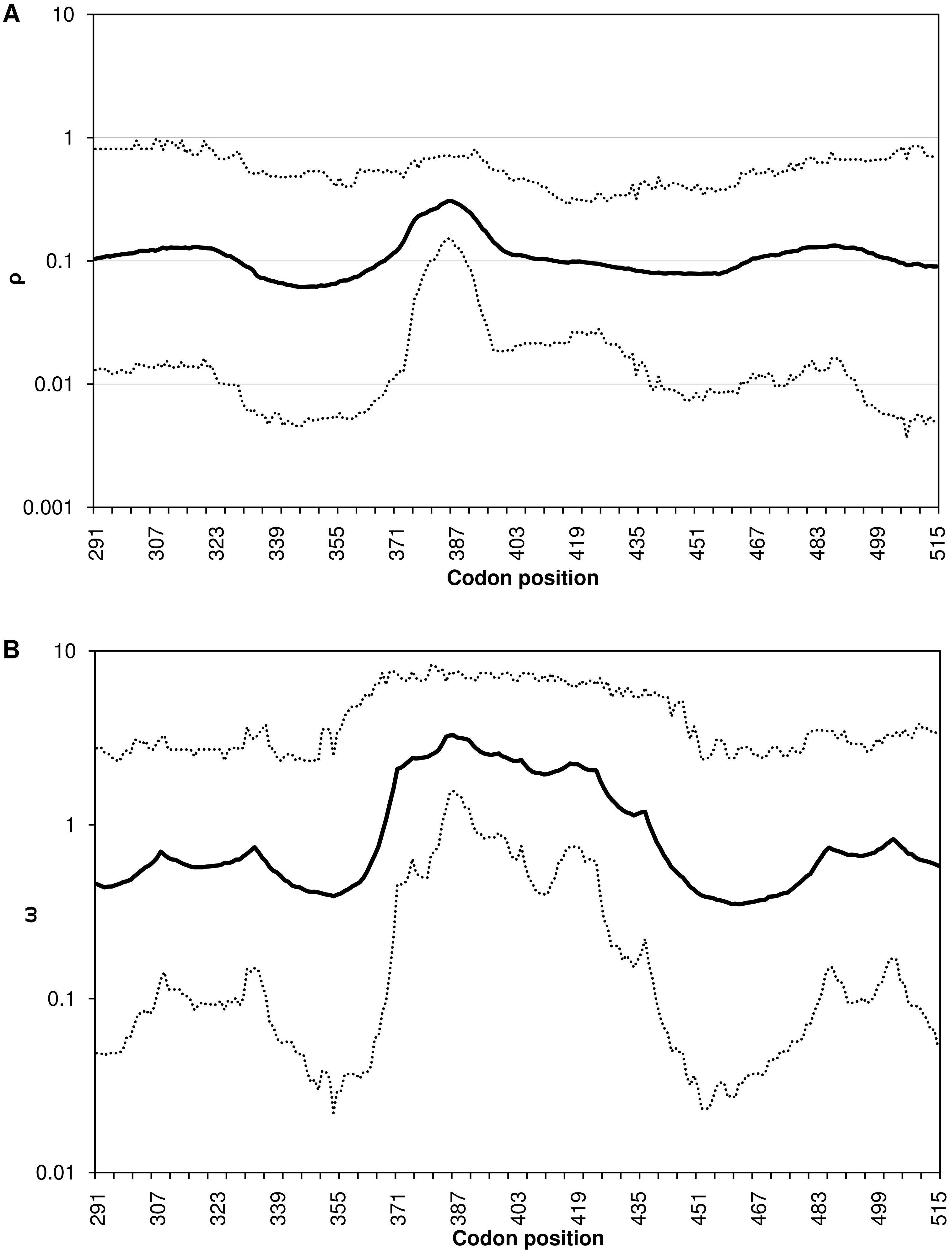

Supplement: Additional file 3 — Recombination and selection parameters in PvDBPII with an alternative set of priors. The spatial variation in recombination and selection parameters across PvDBPII was obtained using omegaMap software. The analysis was performed using an alternative set of priors: μ = Exponential mean 0.02; κ = Exponential mean 2.0; ϕ = Exponential mean 0.1; ω = Exponential mean 1.0; ρ = Exponential Mean 0.14. The means for the priors for μ, κ and ρ were chosen using the posterior distributions generated with the first prior set. (A) Recombination parameter estimates (ρ). The sitewise mean (solid line) and 95% HPD (highest posterior density) interval (dotted lines) are shown. (B) Omega parameter estimates (ω). The sitewise mean (solid line) and 95% HPD intervals (dotted lines) are shown. [file 1475-2875-9-334-S3.TIFF]

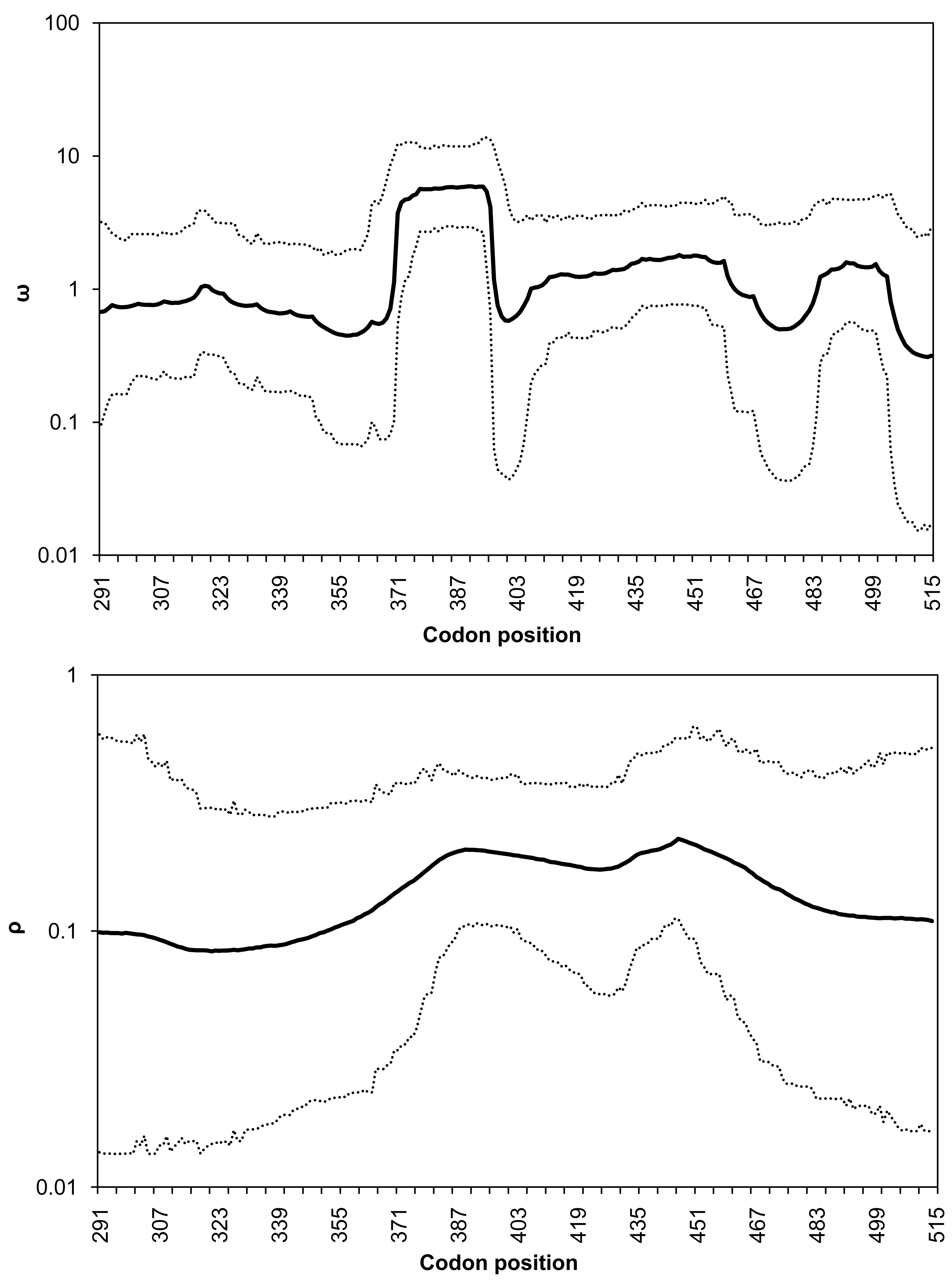

Supplement: Additional file 4 — Spatial variation in recombination (ρ) and omega (ω) among Papuan New Guinea PvDBPII sequences calculated using omegaMap. (A) Evidence of recombination is ρ > 0.1. (B) Evidence of diversifying natural selection is ω > 1 and for purifying natural selection it is ω < 1. The sitewise mean (solid line) and 95% HPD intervals (dotted lines) are shown. [file 1475-2875-9-334-S4.TIFF]
