# Supplementary material for: Genetic variability and natural selection at the ligand domain of the Duffy binding protein in brazilian Plasmodium vivax populations
Source: Malar J. 2010 Nov 22;9:334. doi: 10.1186/1475-2875-9-334 (PMC3003673; doi:10.1186/1475-2875-9-334)
Supplement: Additional file 2 — PvDBPII polymorphisms of Brazilian isolates. This file provides descriptions of the PvDBPII polymorphisms identified in the Brazilian isolates by geographic region as well as polymorphisms that were previously described in other localities. [file 1475-2875-9-334-S2.DOC]

| **Table S1. Description of PvDBP_II_ polymorphisms identified in Brazilian isolates** | | | | | | | | | | | | | | | | | | | | |
| --- | --- | --- | --- | --- | --- | --- | --- | --- | --- | --- | --- | --- | --- | --- | --- | --- | --- | --- | --- | --- |
| **Polymorphisms** | **Nucleotide^1^** | **915** | **924** | **997** | **1111** | **1123** | **1134** | **1151** | **1153** | **1156/**  **1158** | **1169** | **1192** | **1211** | **1251** | **1257** | **1270** | **1309** | **1392** | **1456** | **1508** |
|  |  | AA**T^2^**  AA**C** | AG**G** AG**T** | **C**TT **T**TT | **A**AA**G**AA | **A**AT**G**AT | CG**C** CG**T** | G**G**T G**A**T | **G**AA **A**AA | **A**A**G**  **A**A**T C**A**G** | C**A**T C**G**T | **T**CT**A**CT | A**C**A A**G**A | AA**T** AA**A** | AT**A**AT**G** | **T**TA  **A**TA | **T**GG **C**GG | AT**C**AT**A** | **C**AA **G**AA | A**T**A A**A**A |
|  | **AA** | **305** | **308** | **333** | **371** | **375** | **378** | **384** | **385** | **386** | **390** | **398** | **404** | **417** | **419** | **424** | **437** | **464** | **486** | **503** |
|  |  | N/N | R/S | L/F | K/E | N/D | R/R | G/D | E/K | K/N/Q | H/R | S/T | T/R | N/K | I/M | L/I | W/R | I/I | Q/E | I/K |
| **Region (N)^3^** | **Haplotype** |  |  |  |  |  |  |  |  |  |  |  |  |  |  |  |  |  |  |  |
| **MT(1)/RO(2)** | **1** |  | ..T |  |  |  | ..T |  | A.. | ..T |  | A.. | .G. |  |  | A.. |  |  |  |  |
| **MT(1)** | **2** |  | ..T |  |  |  | ..T |  | A.. | ..T |  | A.. | .G. |  |  | A.. |  |  | G.. |  |
| **MT(1)** | **3** |  |  |  |  |  | ..T |  | A.. | ..T |  | A.. | .G. |  |  | A.. |  |  |  |  |
| **AC(1)/RO(1)** | **4** |  |  | T.. |  | G.. | ..T |  | A.. | ..T |  |  |  | ..A |  | A.. | C.. |  |  | .A. |
| **AC(4)/RO(1)** | **5** |  |  | T.. |  | G.. | ..T |  | A.. | ..T |  |  |  | ..A |  | A.. | C.. |  |  |  |
| **AC(3)/RO(1)** | **6** |  |  |  |  |  | ..T |  | A.. | ..T |  |  |  |  |  |  | C.. |  |  | .A. |
| **AM(1)/RO(2)** | **7** |  |  |  | G.. |  |  |  | A.. | ..T | .G. |  |  | ..A |  | A.. | C.. |  |  | .A. |
| **AM(2)** | **8** |  | ..T |  | G.. |  |  |  | A.. | ..T | .G. |  |  | ..A |  | A.. | C.. |  |  | .A. |
| **AC(1)/RO(1)** | **9** |  |  |  | G.. |  |  |  | A.. | ..T | .G. |  |  | ..A |  | A.. | C.. |  |  |  |
| **RO(1)** | **10** |  |  |  |  |  |  |  | A.. | ..T | .G. |  |  | ..A |  | A.. | C.. |  |  |  |
| **RO(1)** | **11** |  |  |  |  |  |  |  |  | ..T |  |  |  | ..A |  | A.. | C.. |  |  | .A. |
| **RO(1)** | **12** |  |  |  |  |  |  |  |  | ..T | .G. |  |  |  |  |  | C.. |  |  | .A. |
| **AC(1)** | **13** |  |  |  |  |  |  | .A. |  | ..T | .G. |  |  |  |  |  | C.. |  |  | .A. |
| **AM(2)/PA(5)** | **14** |  |  |  |  |  |  |  |  |  |  |  |  |  |  |  |  |  | G.. |  |
| **AP(7)** | **15** | ..C |  |  |  |  |  |  |  |  |  |  |  |  |  |  |  |  | G.. |  |
| **AP(2)/MT(1)/**  **RO(1)** | **16** |  |  |  |  |  |  |  |  |  |  |  |  |  |  |  |  |  |  |  |
| **AP(1)** | **17** |  |  |  | G.. |  |  |  |  |  |  |  |  |  |  |  |  |  |  |  |
| **AM(1)** | **18** |  | ..T |  |  |  |  |  |  |  |  |  |  |  |  |  |  |  |  | .A. |
| **AC(5)/AP(1)/**  **AM(9)/MT(6)/**  **PA(1)/RO(1)** | **19** |  |  |  |  |  |  |  |  |  |  |  |  |  |  |  |  |  |  | .A. |
| **MT(1)** | **20** |  |  |  |  |  |  |  |  |  | .G. |  |  |  |  |  |  |  |  | .A. |
| **AC(2)/MT(2)** | **21** |  |  |  |  |  |  | .A. |  |  | .G. |  |  |  |  |  |  |  |  | .A. |
| **AC(2)/AP(1)/**  **AM(2)/MT(1)/**  **PA(1)/RO(1)** | **22** |  |  |  |  |  |  | .A. |  |  | .G. |  |  |  |  |  |  |  |  |  |
| **MT(1)** | **23** |  |  |  |  |  |  |  |  |  | .G. |  |  |  |  | A.. |  |  |  |  |
| **MT(1)** | **24** |  |  |  | G.. |  |  |  |  |  | .G. |  |  |  |  |  | C.. |  |  |  |
| **MT(1)** | **25** |  |  |  |  |  |  |  |  |  | .G. |  |  |  |  |  | C.. |  |  |  |
| **PA(2)** | **26** |  |  |  | G.. |  |  |  |  | C.. | .G. |  |  |  |  | A.. | C.. |  |  | .A. |
| **PA(1)** | **27** |  |  |  | G.. |  |  |  |  | C.. | .G. |  |  |  |  | A.. | C.. |  |  |  |
| **PA(3)** | **28** |  |  |  |  |  |  |  |  | C.. | .G. |  |  | ..A |  | A.. | C.. |  |  |  |
| **AC(2)** | **29** |  |  |  |  |  |  | .A. |  |  | .G. |  |  | ..A |  | A.. | C.. |  |  |  |
| **AC(3)/AM(3)/**  **PA(1)/RO(1)** | **30** |  |  |  |  |  |  | .A. |  |  | .G. |  |  | ..A |  | A.. | C.. |  |  | .A. |
| **RO(1)** | **31** |  |  |  | G.. |  |  |  |  |  | .G. |  |  | ..A |  | A.. | C.. |  |  |  |
| **AC(1)/MT(1)/**  **RO(2)** | **32** |  |  |  | G.. |  |  |  |  |  | .G. |  |  | ..A | ..G | A.. | C.. |  |  |  |
| **MT(1)** | **33** |  | ..T |  | G.. |  |  |  |  |  | .G. |  |  | ..A | ..G | A.. | C.. | ..A |  |  |
| **AP(2)/MT(6)/**  **PA(4)/RO(2)** | **34** |  |  |  | G.. |  |  |  |  |  | .G. |  |  | ..A | ..G | A.. | C.. | ..A |  |  |
| Other localities^4^ |  | - | PNG | PNG | PNG | PNG | PNG | PNG | PNG | PNG | PNG | - | - | PNG | - | PNG | PNG | - | - | PNG |
|  |  | - | - | - | COL | - | - | COL | COL | COL | COL | - | - | COL | - | COL | COL | - | - | COL |
|  |  | - | THAI | THAI | THAI | THAI | THAI | THAI | THAI | THAI | THAI | THAI | THAI | THAI | THAI | THAI | THAI | - | THAI | THAI |
|  |  | - | - | - | SK | - | - | SK | - | SK | SK | - | - | SK | - | SK | SK | - | - | SK |
|  |  | - | Oth | - | Oth | - | Oth | Oth | Oth | Oth | Oth | Oth | Oth | Oth | Oth | Oth | Oth | Oth | - | Oth |
|  |  | - | SLK | - | SLK | - | SLK | SLK | SLK | SLK | SLK | SLK | SLK | SLK | SLK | SLK | SLK | SLK | SLK | SLK |
|  |  | - | IRAN | - | IRAN | - | IRAN | IRAN | IRAN | IRAN | IRAN | IRAN | IRAN | IRAN | IRAN | IRAN | IRAN | IRAN | IRAN | IRAN |

^1^ Nucleotide and amino acid (AA) numbers according to SAL-1 sequence [39]

^2^ First codon corresponds to the most frequent among Brazilian isolates and the others to the polymorphic ones observed in Brazilian isolates, substitutions are shown in bold.

^3^ Collection States (N=number of individuals): MT – Mato Grosso; AC – Acre; AM – Amazonas; PA – Pará; RO - Rondônia and AP – Amapá

^4^ Other localities where the polymorphisms were also previously identified: Col – Colombia, IRAN – Iran, PNG – Papua New Guinea, SLK – Sri Lanka, SK – South Korea, Oth (others) - Bangladesh, India, Indonesia, Honduras, Mauritania or Vietnam
